# Functional Analysis of *FgNahG* Clarifies the Contribution of Salicylic Acid to Wheat (*Triticum aestivum*) Resistance against Fusarium Head Blight

**DOI:** 10.3390/toxins11020059

**Published:** 2019-01-22

**Authors:** Peng-Fei Qi, Ya-Zhou Zhang, Cai-Hong Liu, Qing Chen, Zhen-Ru Guo, Yan Wang, Bin-Jie Xu, Yun-Feng Jiang, Ting Zheng, Xi Gong, Cui-Hua Luo, Wang Wu, Li Kong, Mei Deng, Jian Ma, Xiu-Jin Lan, Qian-Tao Jiang, Yu-Ming Wei, Ji-Rui Wang, You-Liang Zheng

**Affiliations:** 1 State Key Laboratory of Crop Genetics of Disease Resistance and Disease Control, Chengdu 611130, China; 2 Triticeae Research Institute, Sichuan Agricultural University, Chengdu 611130, China; zhangyazhou@stu.sicau.edu.cn (Y.-Z.Z.); rainbow@stu.sicau.edu.cn (C.-H.L.); qingchen83@sicau.edu.cn (Q.C.); guozhenru@stu.sicau.edu.cn (Z.-R.G.); wyan810@163.com (Y.W.); binjiexu@outlook.com (B.-J.X.); jiangyunfeng2018@163.com (Y.-F.J.); tingzheng@sicau.edu.cn (T.Z.); xigong@stu.sicau.edu.cn (X.G.); luocuihua@stu.sicau.edu.cn (C.-H.L.); wuwang@stu.sicau.edu.cn (W.W.); kongli@sicau.edu.cn (L.K.); dengmei105@163.com (M.D.); jianma@sicau.edu.cn (J.M.); lanxiujin@163.com (X.-J.L.); qiantaojiang@sicau.edu.cn (Q.-T.J.); jirui.wang@gmail.com (J.-R.W.); ylzheng@sicau.edu.cn (Y.-L.Z.)

**Keywords:** salicylic acid, hydroxylase, catechol, mycotoxin, defense

## Abstract

Salicylic acid (SA) is a key defense hormone associated with wheat resistance against Fusarium head blight, which is a severe disease mainly caused by *Fusarium graminearum*. Although *F. graminearum* can metabolize SA, it remains unclear how this metabolic activity affects the wheat–*F. graminearum* interaction. In this study, we identified a salicylate hydroxylase gene (*FG05_08116*; *FgNahG*) in *F. graminearum*. This gene encodes a protein that catalyzes the conversion of SA to catechol. Additionally, FgNahG was widely distributed within hyphae. Disrupting the *FgNahG* gene (Δ*FgNahG*) led to enhanced sensitivity to SA, increased accumulation of SA in wheat spikes during the early infection stage and inhibited development of head blight symptoms. However, *FgNahG* did not affect mycotoxin production. Re-introducing a functional *FgNahG* gene into the Δ*FgNahG* mutant recovered the wild-type phenotype. Moreover, the expression of *FgNahG* in transgenic *Arabidopsis thaliana* decreased the SA concentration and the resistance of leaves to *F. graminearum*. These results indicate that the endogenous SA in wheat influences the resistance against *F. graminearum*. Furthermore, the capacity to metabolize SA is an important factor affecting the ability of *F. graminearum* to infect wheat plants.

## 1. Introduction

Salicylic acid (SA) is a key signaling molecule for regulating plant resistance to diverse pathogens. It triggers systemic acquired resistance (SAR) and induces the expression of a set of genes encoding pathogenesis-related (PR) proteins as well as the accumulation of H_2_O_2_ [1,2]. The biosynthesis of SA in plants mainly involves the phenylalanine ammonia lyase (PAL) pathway and the isochorismate synthase (ICS) pathway [3,4]. Both pathways use the same primary metabolite, chorismate [3,5,6], which can be converted to SA through benzoate intermediates or coumaric acid in a series of enzymatic reactions involving PAL [7,8]. The ICS pathway is responsible for the biosynthesis of more than 95% of SA and regulates the defense responses of *Arabidopsis thaliana*, *Nicotiana benthamiana* and barley against fungal infections by manipulating the accumulation of SA [6,9,10].

**Figure 1:**
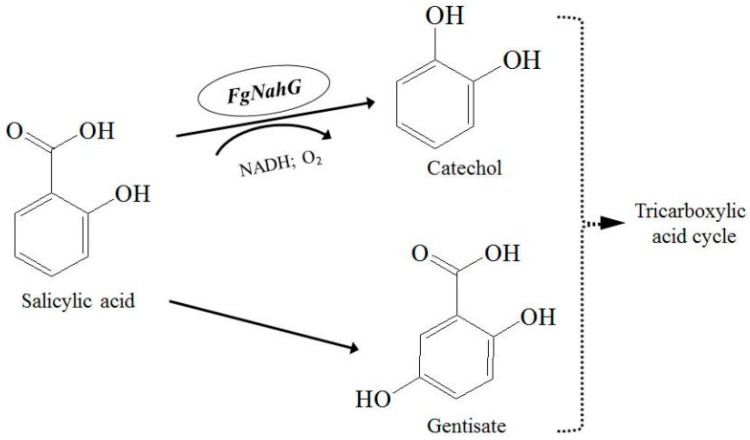
Possible salicylic acid (SA) metabolic pathways in *Fusarium graminearum* [11,16]. The dotted arrows indicate a series of enzymatic reactions that have not been fully characterized.

To counteract the ability of plants to produce SA in response to pathogens, some pathogenic fungi can degrade SA [11,12]. These fungi can convert SA to catechol and/or gentisate directly or through intermediates. Catechol and gentisate are then metabolized in the tricarboxylic acid cycle (Figure 1). Salicylate hydroxylase catalyzes SA to catechol and the corresponding genes have been identified in fungi. The expression of a cytoplasmic salicylate hydroxylase gene (*Shy1*) in *Ustilago maydis* can be activated on an artificial medium with SA as the sole carbon source but is not required for virulence during the infection of maize seedlings [13]. The protein encoded by *Efe-shyA* in the endophytic fungus *Epichloë festucae* exhibits salicylate hydroxylase activity but it does not inhibit host plant defenses [14]. The genome of *Candidatus Liberibacter asiaticus* (*Ca. L. asiaticus*), which is the pathogen responsible for Huanglongbing disease, has an actively expressed salicylate hydroxylase gene, *SahA*, which is required for degrading SA and suppressing the citrus plant defense responses [15]. It is likely that only some fungal salicylate hydroxylase genes encode virulence factors.

*Fusarium graminearum*, which can metabolize SA via the catechol and/or gentisate pathways [13], is one of the most destructive *Fusarium* species and is responsible for Fusarium head blight (FHB) in wheat and other small cereal grains [17,18]. Outbreaks of FHB result in huge economic losses due to decreased yield and the contamination of grains by mycotoxins that are harmful to humans and animals [19,20]. The contribution of SA to the interaction between wheat and *F. graminearum* remains unclear. However, previous studies revealed that the infection of wheat heads by *F. graminearum* stimulates the considerable accumulation of SA and the expression of SA-related genes [21,22]. Moreover, SA significantly and directly affects *F. graminearum* by inhibiting mycotoxin production, conidial germination and mycelial growth [16] by downregulating the expression of the chitin synthase gene *FgCHS8* and the *cis*-12 linoleic acid isomerase gene *FgLAI12* [23,24]. Deletion of the SA exporter gene *FgABCC9* results in increased sensitivity to SA, decreased accumulation of the mycotoxin deoxynivalenol (DON) and fewer blight symptoms in wheat [25]. These results strongly suggest that SA helps protect wheat from *F. graminearum*. Additionally, treating wheat roots and leaves with SA/MeSA (methyl salicylate) can enhance wheat resistance against *F. graminearum* [26,27]. However, treating wheat spikes with exogenous SA reportedly does not improve wheat FHB resistance [16,28], possibly because *F. graminearum* can efficiently metabolize SA [16]. Unexpectedly, disrupting the salicylate hydroxylase gene *FGSG_03657* has no effect on *F. graminearum* virulence [29]. Considering the importance of SA for wheat FHB resistance and the efficiency with which *F. graminearum* degrades SA, we hypothesized that there is an unidentified salicylate hydroxylase gene in the *F. graminearum* genome.

In this study, we identified a new salicylate hydroxylase gene in *F. graminearum* (*FG05_08116*; *FgNahG*). The expression of this gene was upregulated by SA when SA was the sole carbon source. Moreover, the effects of mutations to *FgNahG* were analyzed to elucidate the function of the encoded protein during SA degradation as well as the role of SA in the wheat–*F. graminearum* interaction. The results of this study may be useful for developing novel strategies to control FHB.

## 2. Results

### 2.1. Deletion and Complementation of FgNahG in F. graminearum

**Figure 2:**
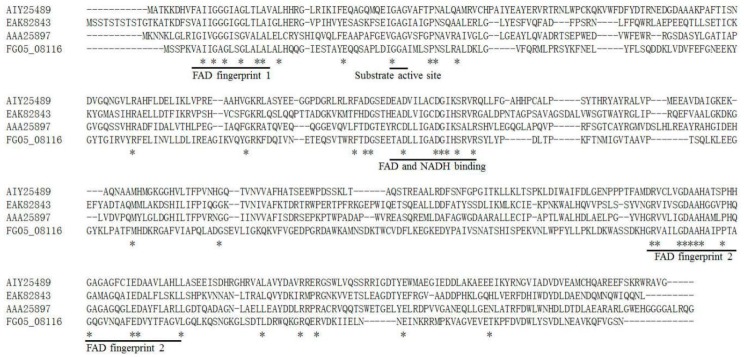
Alignment of the deduced amino acid sequences encoded by salicylate hydroxylase genes from *Epichloë festucae* (accession No. AIY25489, [14]), *Ustilago maydis* (EAK82843, [13]), *Pseudomonas putida* (AAA25897, [30]) and *F. graminearum* (Fg05_08116). The underlined sequences indicate the conserved domains. FAD, Flavin adenine dinucleotide; NADH, Nicotinamide adenine dinucleotide. Asterisks indicate the residues that are present in all sequences.

**Figure 3:**
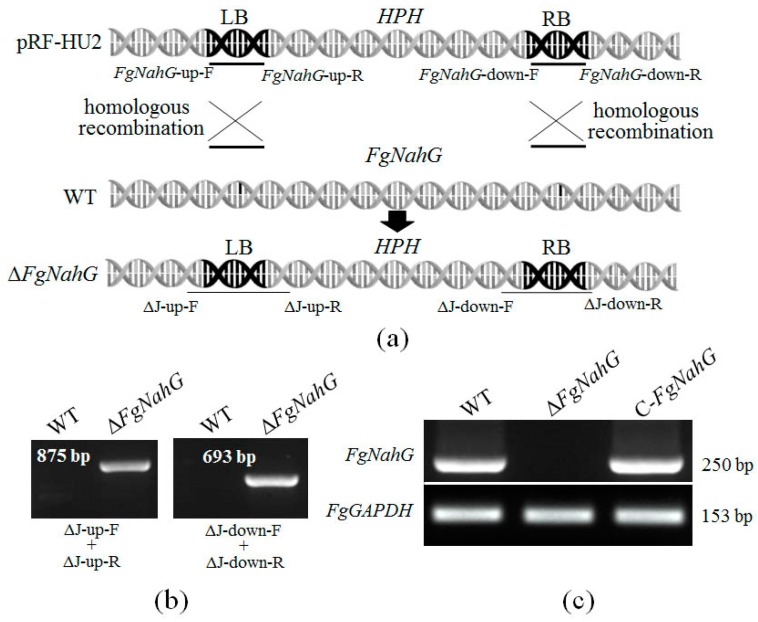
Construction of *FgNahG* deletion and complementation mutants. (**a**) The left border (LB) and right border (RB) were amplified from the wild-type (WT) strain with the *FgNahG*-up-F/*FgNahG*-up-R and *FgNahG*-down-F/*FgNahG*-down-R primer pairs, respectively, for the subsequent construction of recombinant plasmids. The Δ*FgABCC9* mutants were generated by the homologous recombination between the recombinant plasmid and the *FgNahG* sequence. The bold fragments correspond to the LB and RB of *FgNahG*. *HPH*, hygromycin B phosphotransferase gene. (**b**) Verification of Δ*FgNahG* by PCR with the ΔJ-up-F/ΔJ-up-R and ΔJ-down-F/ΔJ-down-R primer pairs. (**c**) Verification of the expression of *FgNahG* in Δ*FgNahG* and C-*FgNahG* strains by RT-PCR with the Rj-*FgNahG*-F/Rj-*FgNahG*-R primer pair, which targeted the *FgNahG* coding region. *FgGAPDH* was used as a reference gene. All PCR products were verified by sequencing at a commercial company (Qingke, Chengdu, China).

The *FgNahG* gene (*FG05_08116*) is intronless and the encoded protein contains conserved domains related to SA degradation (Figure 2), indicating that it is a putative salicylate hydroxylase. To functionally characterize *FgNahG* in *F. graminearum*, 10 Δ*FgNahG* mutants were generated by homologous recombination and verified by sequencing (Figure 3a,b). To create complementation mutants (C-*FgNahG*), *FgNahG* was randomly inserted into the genome of the Δ*FgNahG* mutants. Eight C-*FgNahG* mutants were used throughout this study. The Δ*FgNahG* and C-*FgNahG* mutants were further verified by reverse transcription PCR (RT-PCR). As expected, *FgNahG* was not expressed in the Δ*FgNahG* strain but was expressed in the C-*FgNahG* strain (Figure 3c).

### 2.2. Influence of FgNahG on Mycelial Growth

**Figure 4:**
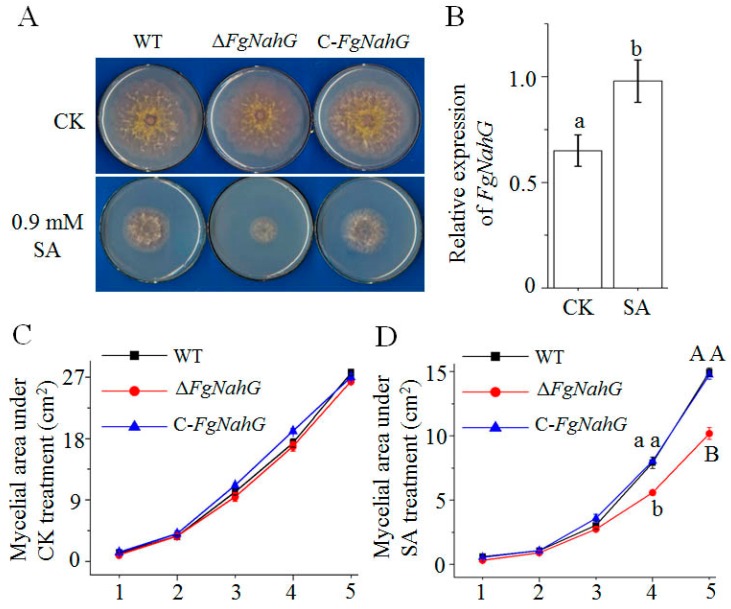
Effect of *FgNahG* on mycelial growth. (**A**) Mycelial growth of the wild-type (WT), Δ*FgNahG* and C-*FgNahG* strains on mSNA plates with and without SA at 5 days after inoculations with 1 × 10^3^ *F. graminearum* conidia. Five biological replicates were analyzed per treatment. (**B**) Comparison of *FgNahG* expression in WT hyphae under the same conditions as the mycelial growth experiment presented in panel (**A**). (**C**) Comparison of the mycelial area under CK treatment as panel (**A**) during 1–5 days after initial inoculation. (**D**) Comparison of the mycelial area under SA treatment as panel (**A**) during 1–5 days after initial inoculation. CK, control treatment; SA, salicylic acid treatment. Different lower-case and capital letters above each column indicate significant differences at *p* ≤ 0.05 and *p* ≤ 0.01, respectively.

To evaluate the growth changes caused by the disruption of *FgNahG* in *F. graminearum*, the mycelial growth of the wild-type (WT), Δ*FgNahG* and C-*FgNahG* strains was analyzed (Figure 4A). The SA treatment inhibited the mycelial growth of the WT, Δ*FgNahG* and C-*FgNahG* strains, which is consistent with the results of a previous study [16]. Notably, the Δ*FgNahG* mutant grew slower than the WT and C-*FgNahG* strains following the SA treatment (Figure 4A,D), while the mycelia grew similarly under control conditions (Figure 4A,C). Consistent with its effects on mycelial growth in response to SA, *FgNahG* expression was induced by SA (Figure 4B). These observations implied that *FgNahG* expression helps *F. graminearum* deal with SA stress.

### 2.3. Subcellular Localization

**Figure 5:**
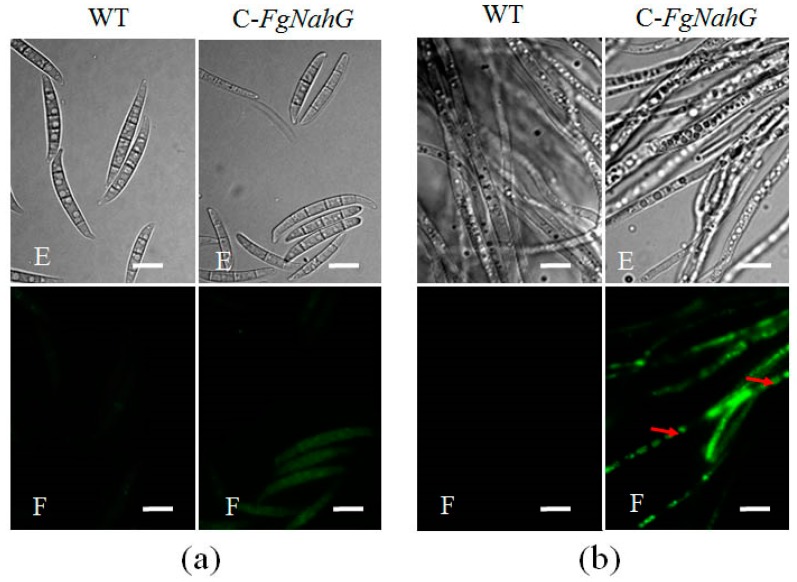
Subcellular localization of the FgNahG protein. (**a**) Conidia of the wild-type (WT) and C-*FgNahG* strains. (**b**) Hyphae of the WT and C-*FgNahG* strains. Red arrows indicate the fluorescent signal. E, optical microscope; F, fluorescence microscope. Scale bar, 10 µm.

The subcellular localization of FgNahG was investigated with the C-*FgNahG* mutant. Specifically, the green fluorescent protein was tagged to the C-terminal of FgNahG in C-*FgNahG*. A subsequent analysis of fluorescence revealed that FgNahG was widely distributed both in conidia (Figure 5a) and in hyphae (Figure 5b), suggesting that FgNahG protects *F. graminearum* from SA in wheat at the subcellular level.

### 2.4. Determination of the FgNahG Enzyme Activity In Vitro

**Figure 6:**
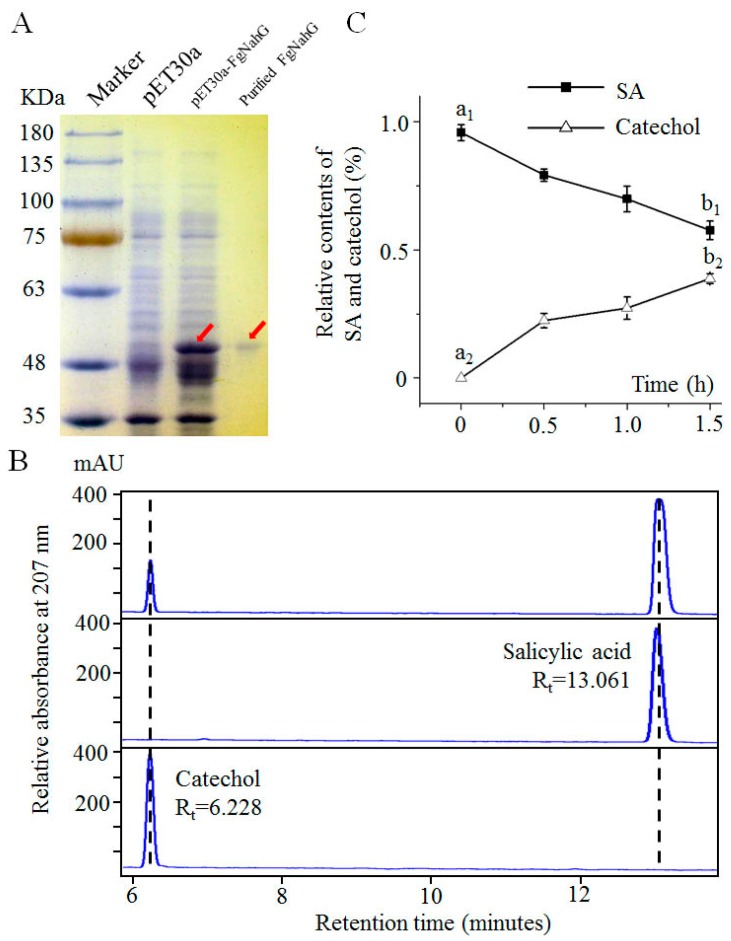
Expression of *FgNahG* in *E. coli* and the activity of the encoded enzyme. (**A**) SDS-PAGE analysis of the FgNahG protein. pET-30a, proteins from *E. coli* cells carrying the pET-30a vector; pET-30a-FgNahG, proteins from *E. coli* cells carrying the recombinant pET-30a-FgNahG vector; purified FgNahG, FgNahG protein purified from pET-30a-FgNahG cells. Red arrows indicate the FgNahG protein. (**B**) HPLC analysis. The lower and middle panels present the peaks for catechol and salicylic acid (SA), respectively. The upper panel reveals the presence of SA and catechol when SA was mixed with recombinant FgNahG protein. Rt, retention time. (**C**) Measurement of the relative SA and catechol contents in the reaction mixture. The SA and catechol concentrations were determined by HPLC at 0, 0.5, 1.0 and 1.5 h after the addition of SA. Analyses at each time point were completed with three biological replicates per treatment. The SA and catechol contents at 0 and 1.5 h were compared. Different letters indicate significant differences at *p* ≤ 0.01.

To test whether FgNahG exhibits salicylate hydroxylase activity as expected, a His-tagged FgNahG protein was heterologously expressed in *Escherichia coli* (Figure 6A). The purified and renatured FgNahG protein was then analyzed in an enzyme assay (Figure 6A), which confirmed the recombinant FgNahG hydroxylated SA to catechol (Figure 6B,C). A rapid increase in catechol abundance and a decrease in SA concentration were observed between 0 and 1.5 h. Moreover, the purified and renatured extracts from the control *E. coli* strain (i.e., carrying the empty vector) did not induce any significant changes in the SA content between 0 and 1.5 h. These results indicated that FgNahG encodes a salicylate hydroxylase that catalyzes the conversion of SA to catechol (Figure 1).

### 2.5. Effect of FgNahG on Fungal Pathogenicity in Wheat

**Figure 7:**
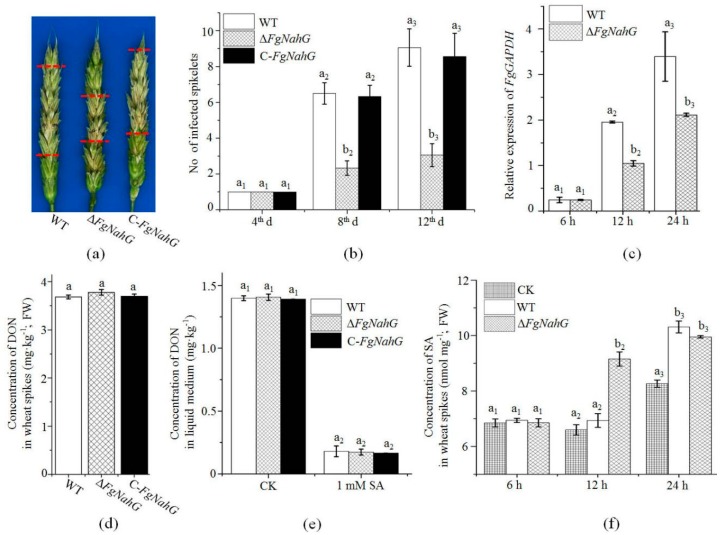
Effect of *FgNahG* on *F. graminearum* pathogenicity in wheat. (**a**) Head blight symptoms of spikes inoculated with the wild-type (WT), Δ*FgNahG* or C-*FgNahG* strains at 12 days after inoculations. Red lines indicate the spread of head blight symptoms in spikes. (**b**) Numbers of infected and bleached spikelets at 4, 8 and 12 days after inoculations. (**c**) Relative expression of *FgGAPDH* in wheat spikes at 6, 12 and 24 h after the initial inoculations. (**d**) Comparison of deoxynivalenol (DON) contents in wheat spikes inoculated with the WT, Δ*FgNahG* or C-*FgNahG* strains at 6 days after inoculations. (**e**) Measurement of DON production by mycelia grown in liquid medium (the same amount of mycelia was used). The DON production data are provided as mg kg^−1^ mycelia. (**f**) Levels of SA in spikes inoculated with water (CK treatment), the WT strain or the Δ*FgNahG* strain at 6, 12 and 24 h after inoculations. Values are provided as the mean ± standard deviation of three biological replicates per treatment. Different letters above each column indicate significant differences at *p* ≤ 0.05. FW, fresh weight.

To clarify whether *FgNahG* influences pathogenicity during infections of wheat, head blight symptoms in the spikes inoculated with conidial suspensions of WT, Δ*FgNahG* or C-*FgNahG* strains were compared. There were fewer disease symptoms and less fungal biomass in the spikes inoculated with the Δ*FgNahG* mutant than in the spikes inoculated with WT or C-*FgNahG* strains (Figure 7a–c). Additionally, an analysis of DON contents in liquid medium and wheat spikes revealed that the Δ*FgNahG* mutant produced the same amount of DON as the WT and C-*FgNahG* strains (Figure 7d,e), even after 1 mM SA was added to the liquid medium (Figure 7e).

Considering that *FgNahG* encodes a salicylate hydroxylase, the SA contents in spikes inoculated with water (control), the WT strain or the Δ*FgNahG* mutant were compared at 6, 12 and 24 h post-inoculation (hpi; Figure 7f). At 6 hpi, no significant differences were observed, suggesting that *F. graminearum* did not stimulate SA biosynthesis in wheat spikes at this stage (conidia are more sensitive to SA than mycelia) [16]. At 12 hpi, spikes inoculated with the Δ*FgNahG* mutant accumulated considerably more SA than the spikes inoculated with water or the WT strain. At 24 hpi, there were no significant differences in the SA levels in spikes inoculated with the Δ*FgNahG* or WT strains (Figure 7f). These results indicated that SA is more important for wheat resistance against *F. graminearum* during the early infection stage and that *FgNahG* protects *F. graminearum* from the endogenous SA in wheat.

Jasmonic acid (JA) interacts with SA in wheat spikes and JA is an important factor in wheat defense against *F. graminearum* [21]. The JA contents in spikes inoculated with water (control), the WT strain or the Δ*FgNahG* mutant were compared at 6, 12 and 24 hpi (Supplementary Figure S1) as well. At 6 hpi, no significant differences were observed. At 12 hpi, spikes inoculated with the Δ*FgNahG* accumulated more JA than the spikes inoculated with water or the WT strain. Interestingly, spikes inoculated with the WT strain accumulated less JA than those inoculated with water. At 24 hpi, there were no significant difference in the JA levels in spikes inoculated with water or WT strain. The JA content in spikes inoculated with the Δ*FgNahG* mutant remained significantly higher than those under control and WT treatments. These results indicated that the earlier induction of JA is more important for wheat defense against *F. graminearum* and that the higher SA contents (Figure 7f) contributes to the accumulation of JA in wheat spikes during the early infection stage.

### 2.6. Expression of FgNahG in Transgenic A. thaliana

**Figure 8:**
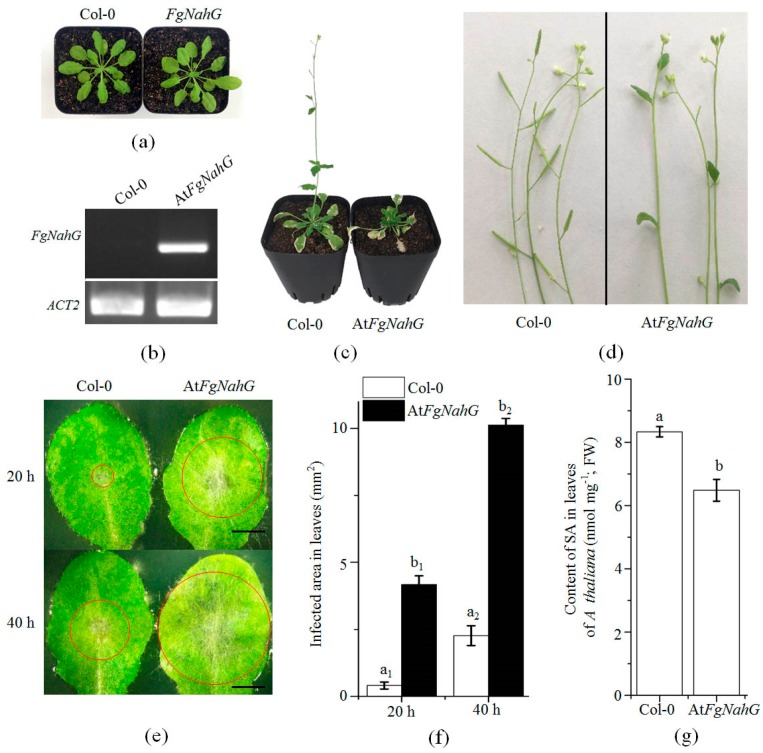
Evaluation of the function of *FgNahG* in *A. thaliana*. (**a**) Wild-type (WT) (*Col-0*) and At*FgNahG* plants at 30 days after germination. (**b**) Verification of *FgNahG* expression in the leaves of WT and At*FgNahG* plants by RT-PCR. The actin 2 gene (*ACT2*, *AT3G18780*) was used as a control [30]. (**c**) Phenotypes of the WT and At*FgNahG* plants at 45 days after germination. (**d**) Inflorescences of WT and At*FgNahG* plants at 60 days after germination. (**e**) Comparison of *F. graminearum*-induced disease symptoms in the leaves of WT and At*FgNahG* plants at 20 and 40 h after inoculations. Scale bar, 1 cm. Red circles indicate the area with disease symptoms. (**f**) Comparison of the leaf areas infected by the WT strain under the same conditions as the inoculation experiment presented in panel (**e**). Analyses were completed with 10 biological replicates per treatment. (**g**) Comparison of the SA contents in the leaves of WT and At*FgNahG* plants at 30 days after germination. Analyses were completed with three biological replicates per treatment. Different letters above each column indicate significant differences at *p* ≤ 0.05.

To confirm its function, *FgNahG* was expressed in *A. thaliana* and 12 positive T_2_ lines were examined (Figure 8a). The RT-PCR results confirmed that *FgNahG* was expressed normally in transgenic *A. thaliana* plants (At*FgNahG*) (Figure 8b). At 30 days after germination, there were no obvious differences between WT (*Col-0*) and At*FgNahG* plants regarding plant architecture under short-day conditions (Figure 8a). However, under long-day conditions (to induce reproductive growth), At*FgNahG* plants exhibited enhanced leaf senescence and delayed flowering compared with WT plants at 45 and 60 days after germination (Figure 8c,d). As expected, the expression of *FgNahG* in *A. thaliana* decreased the accumulation of SA in leaves (Figure 8g) and ultimately decreased the resistance of the transgenic plants to *F. graminearum* (Figure 8e,f).

## 3. Discussion

Salicylic acid is an important phytohormone that regulates many physiological and biochemical processes throughout the plant lifespan [31,32]. Consistent with the phenotypes of the At*FgNahG* lines analyzed in this study (Figure 8c,g), the overexpression of a bacterial *NahG* gene reportedly decreases SA levels, delays flowering and increases leaf senescence in *A. thaliana* [33,34]. Similarly, SA is necessary to induce flowering in *Pharbitis nil* [35]. These observations and our enzyme assay data (Figure 6B,C) indicate that *FgNahG* encodes a salicylate hydroxylase.

Salicylic acid is important for FHB resistance in wheat. Earlier investigations confirmed that an infection by *F. graminearum* increases SA accumulation and activates SA signaling in wheat spikes [21,22,36,37]. Meanwhile, in barley, *ICS* overexpression increases the SA concentration, while also repressing *F. graminearum* infections at 24 and 48 hpi but not at later infection stages [10]. Additionally, SA adversely affects the cell wall and cell membrane of *F. graminearum* by downregulating the expression levels of the fungal class VIII chitin synthase gene (*FgCHS8*) and the *cis*-12 linoleic acid isomerase gene (*FgLAI12*) [23,24], which may inhibit conidial germination and mycelial growth [16]. However, the application of exogenous SA to wheat heads reportedly does not enhance wheat FHB resistance, even at very high concentrations [16,28]. Considering the toxicity of SA, it was previously speculated that *F. graminearum* can decrease the endogenous SA content of wheat below the toxicity threshold by exporting and degrading SA [16]. As expected, deletion of the SA exporter gene *FgABCC9* results in increased sensitivity to SA and fewer head blight symptoms in wheat [25]. Analyses of Δ*FgNahG* mutants revealed that SA contributes to the resistance of wheat against *F. graminearum* and that *FgNahG* expression enables *F. graminearum* to overcome the toxicity of endogenous SA in wheat.

Salicylic acid is especially important for wheat defenses against *F. graminearum* during the early infection stage. *Fusarium graminearum* has a brief biotrophic-like relationship with its host before switching to the necrotrophic phase [38]. Additionally, SA-mediated defense responses are associated with resistance against biotrophic and hemibiotrophic pathogens [39]. Ding et al. [36] and Ameye et al. [27] proposed that induced SA production very early during an infection influences the mechanism that protects wheat against *F. graminearum*. We compared the SA contents in spikes inoculated with the WT strain or Δ*FgNahG* mutant (Figure 7f). Notably, SA contents increased in response to the Δ*FgNahG* mutant as early as 12 hpi, indicating the importance of *FgNahG* for lowering the amount of endogenous SA in wheat during the early infection stage.

Only some salicylate hydroxylase genes in fungi are required for suppressing host defenses. Besides *FgNahG* in *F. graminearum*, *SahA*, which encodes a salicylate hydroxylase in Ca. *L. asiaticus*, can inhibit plant defense responses [15]. Qi et al. [16] identified *FGSG_03657* as a candidate salicylate hydroxylase gene for degrading SA in *F. graminearum*; this gene is needed for growth in cultures under SA stress conditions. Rocheleau et al. [29] proved that disrupting the salicylate hydroxylase gene *FGSG_03657* has no effect on fungal virulence during infections of wheat. Meanwhile, *Shy1*, which is a salicylate hydroxylase gene in *U. maydis*, is necessary for growth on media with SA as the sole carbon source but is not required for virulence [13]. Similarly, in the cool-season grass endophytic fungus *E. festucae*, *Efe-shyA* encodes a salicylate hydroxylase that is not required for overcoming plant defenses [14]. Why salicylate hydroxylase does not influence or negligibly contributes to virulence on host plants under certain conditions has not been established, although there are several possible explanations, including the following six: (1) The amount of salicylate hydroxylase produced by the pathogen is insufficient to maintain the SA level at the infection site below the toxicity threshold; (2) The subcellular location of the enzyme is restricted within fungal cells, resulting in excessive SA concentrations in most fungal cell parts; (3) The expression level of some salicylate hydroxylase genes is too low in the absence of SA. Infections by pathogens induce a rapid accumulation of SA around infection sites. Consequently, SA considerably damages pathogens before they can produce enough salicylate hydroxylase; (4) Some proteins/chemicals released by host plants can efficiently inhibit salicylate hydroxylase activity during infections, even if salicylate hydroxylase genes are highly expressed; (5) Salicylic acid and SA-induced defense responses play a minor role in defenses against certain pathogens; (6) Some unknown genes/pathways play a more important role in SA degradation than the examined genes/pathways. For example, although both *FGSG_03657* and *FgNahG* are actively expressed salicylate hydroxylase genes in *F. graminearum*, *FgNahG* was not identified in a previous transcriptome analysis under SA stress conditions in culture [16]. *Fusarium graminearum* has at least two actively expressed salicylate hydroxylase genes that encode enzymes that efficiently degrade SA [16]. Additionally, FgNahG is widely distributed in hyphae. The *FgNahG* gene was highly expressed on an artificial medium in the absence of SA (Figure 4B) and its expression was upregulated by less than 1-fold when SA was the sole carbon source. Moreover, FgNahG efficiently degraded SA in spikes (Figure 7f). Previous studies revealed that SA contributes to wheat FHB resistance [16,23,24,25,26,27]. Therefore, the fact that *FgNahG* is required for the virulence of *F. graminearum* on wheat is not surprising (Figure 6).

In this study, we observed that *FgNahG* was expressed normally in the absence of SA (Figure 4B), while deleting *FgNahG* from the *F. graminearum* genome did not affect fungal growth in the absence of exogenous SA (Figure 4A). Thus, *FgNahG* is not essential for fungal growth when there is a lack of SA. The constitutive expression of *FgNahG* in the absence of SA and the upregulated expression induced by SA (Figure 4B) reflect the selection pressure of SA produced by host plants during the evolution of *F. graminearum*.

The contamination of grains by mycotoxins, such as DON, is the most serious problem caused by FHB. SA dramatically inhibited DON production in *F. graminearum* (Figure 7e), while SA induced the expression of *FgNahG* (Figure 4B). Furthermore, the lack of *FgNahG* did not affect DON accumulation in the liquid medium with or without SA (the same mycelial amount was used; Figure 7e). It is obvious that upregulated and downregulated *FgNahG* expression levels had no effect on DON production. Consequently, although SA significantly regulates DON contents under culture conditions, *FgNahG* is not involved.

Interestingly, the Δ*FgNahG* mutant produced the same amount of DON as the WT and C-*FgNahG* strains in wheat spikes (Figure 7d), even though the fungal biomass was lower (Figure 7c) and the SA content was higher (Figure 7f) in spikes inoculated with the Δ*FgNahG* mutant. Therefore, the Δ*FgNahG* mutant produced more DON than the WT strain during their interactions with wheat spikes, even at relatively high endogenous SA concentrations, relative to the fungal biomass. Similarly, Ameye et al. [27] reported that the priming of wheat with the green-leaf volatile *Z*-3-hexenyl acetate enhances defense responses against *F. graminearum*, while also increasing DON production. In contrast, the plant hormone abscisic acid can enhance wheat susceptibility to *F. graminearum* [21] but decrease the accumulation of DON in spikes (unpublished data). It is obvious that DON production in wheat spikes is due to the interaction between *F. graminearum* and the host. We speculate that some wheat proteins and/or chemicals stimulate DON production in fungi. Therefore, we should be cautious when targeting *FgNahG* while developing novel strategies to control FHB.

## 4. Materials and Methods

### 4.1. Experimental Materials and Growth Conditions

All experiments were conducted with the *F. graminearum* isolate DAOM180378 (Canadian Fungal Culture Collection, AAFC, Ottawa, ON, Canada), which is highly virulent in wheat. Conidia were produced in carboxymethyl cellulose liquid medium at 28 °C, with shaking (180 rpm) for 5 days [40]. The effects of SA on mycelial growth were assessed on mSNA plates supplemented with 0.9 mM SA, as previously described [25]. The growing mycelia on mSNA plates were scanned with the EPSON Perfection V700 Photo Scanner (Seiko Epson, Bekasi, Indonesia) during 1–5 days post-inoculation, after which the mycelial area was measured with the Computer Aided Design program (version 2007, Autodesk, San Rafael, CA, USA). Unless specifically noted, all chemicals were purchased from Sigma-Aldrich (St Louis, MO, USA).

*Triticum aestivum* cultivar ‘Roblin,’ which was used for wheat experiments, is susceptible to *F. graminearum* infections. Wheat seeds were sown in soil fertilized with 15-15-15 (N-P-K) compound fertilizer and the resulting plants were grown in greenhouses under a 16-h day (23 °C)/8-h night (18 °C) cycle. Additionally, *A. thaliana* ecotype *Col-0* plants were also analyzed. Seeds were sown in Petri dishes containing Murashige and Skoog medium solidified with 0.7% (*w/v*) agar. The Petri dishes were incubated at 4 °C for 3 days before being transferred to a growth chamber. The *Col-0* and transgenic plants were grown next to each other in the same tray to minimize possible variations in growth conditions. Plants were grown at 22 °C under short-day conditions (10-h light/14-h dark) for an extended vegetative growth phase of 30 days and then exposed to long-day conditions (16-h light/8-h dark) to induce reproductive growth.

### 4.2. Sequence Analysis and Primer Design

The *FgNahG* nucleotide sequence was downloaded from the Ensembl Fungi database (http://fungi.ensembl.org/index.html). ClustalX was used for multiple sequence alignments [41], while PCR primers were designed with Primer Premier (version 5.0; Premier Biosoft, Palo Alto, CA, USA). Details regarding the primers used in this study are listed in Table 1.

### 4.3. Transformation

The cetyltrimethylammonium bromide (CTAB) method [43] was used to extract genomic DNA from *F. graminearum* mycelia cultured for 5 days at 25 °C on mSNA medium (1 g KH_2_PO_4_, 1 g KNO_3_, 0.5 g MgSO_4_, 0.5 g KCl, 1 g glucose, 1 g sucrose and 20 g agar per liter) in plates. To generate *FgNahG* deletion mutants (Δ*FgNahG*), the pRF-HU2 vector was used for a targeted gene replacement in *F. graminearum* via an *Agrobacterium tumefaciens*-mediated transformation [44,45]. The *FgNahG* sequence was amplified by PCR from the genomic DNA of the WT strain (primers R-*FgNahG*-F and R-*FgNahG*-R) and then ligated into the pCAMBIA1302 vector (pCAMBIA1302-*FgNahG*) (Figure 3a; Table 1). The T-DNA region of pCAMBIA1302-*FgNahG* was inserted into the genome of the Δ*FgNahG* mutant to generate the C*-FgNahG* strain. Additionally, the T-DNA region of pCAMBIA1302-*FgNahG* was inserted into *A. thaliana* according to the floral dip method [46] to produce At*FgNahG* lines. The T_1_ transgenic lines obtained through hygromycin selection (50 μg mL^−1^) were verified by sequencing. Phenotypic analyses were completed with the T_2_ generation and the results were confirmed with the T_3_ generation. All experiments were completed with homozygous plants.

### 4.4. In Vitro Expression of FgNahG in E. coli

The *FgNahG* cDNA sequence was amplified by PCR with the 30a-*FgNahG*-F and 30a-*FgNahG*-R primer pair and ligated into the pET30a vector (with a His tag sequence) for the subsequent production of the FgNahG::His fusion protein. The recombinant vector was inserted into *E. coli* BL21 (DE3) cells (Tiangen, Beijing, China), which were then cultured in Luria-Bertani [47] liquid medium until the optical density at 600 nm reached 0.8. The production of FgNahG was induced by the addition of 0.1 mM isopropyl β-D-1-thiogalactoside and an incubation with shaking (210 rpm) for 12 h at 25 °C. The *E. coli* cells carrying pET30a-*FgNahG* were collected and lysed by sonication in 20 mM phosphate buffer (pH 7.4; with 4 M urea and 500 mM NaCl), after which the His-tagged FgNahG proteins were purified with the His-Bind resin at 4 °C, following the manufacturer’s instructions (Beyotime, Shanghai, China). The purity of the collected FgNahG was assessed by SDS-PAGE [48] and proteins were renatured by dialysis (8000–14,000 Da) in a solution comprising 50 mM Tris-HCl and 2 mM dithiothreitol (pH 8.0) at 4 °C. The concentration of the renatured FgNahG protein was quantified with the Quick Start Bradford Dye Reagent (Bio-Rad, Hercules, CA, USA).

### 4.5. Enzyme Activity Assay

An enzyme assay was completed as described by Ambrose et al. [14] and Bosch et al. [49], with some modifications. The 1-mL reaction mixture (pH 7.4) contained 200 μM SA, 200 mM NADH-Na_2_, 20 μM flavin adenine dinucleotide (disodium salt), 0.4 mM FeSO_4_·7H_2_O, 0.1 mg mL^−1^ catalase, 0.01% H_2_O_2_, 20 μg renatured FgNahG and 10 mM potassium phosphate buffer (pH 7.4). All reactions were initiated by adding FgNahG and stopped by adding 1 mL 50% (*v*/*v*) acetonitrile and heating in boiling water for 1 min. After a centrifugation at 14,000× *g* for 10 min, the supernatant was analyzed by high-performance liquid chromatography. Compounds were separated with the InertsiI^®^ ODS3 C18 column (5 mm, 250 × 4.6 mm, GL Sciences, Hangzhou, China) and the Agilent Technologies 12,000 series HPLC system (Santa Clara, CA, USA). The UV detection was completed at 207 nm with a 0.01 AUFS range. The mobile phase consisted of solvent A (phosphoric acid in water, pH 2.5) and solvent B (acetonitrile), with a solvent gradient as follows: initial 75% A to 25% B for 15 min and then a linear gradient to 45% solvent B from 16 to 21 min, at a flow rate of 1 mL min^−1^.

### 4.6. Microscopy

To analyze hyphae by optical and fluorescence microscopy, 3 mL mSNA liquid medium (1 g KH_2_PO_4_, 1 g KNO_3_, 0.5 g MgSO_4_, 0.5 g KCl, 1 g glucose and 1 g sucrose per liter) was inoculated with 1000 conidia. The medium was then incubated at 28 °C on an orbital shaker (120 rpm) for 48 h in darkness. Conidia produced in carboxymethyl cellulose liquid medium as described above were collected. The conidia and hyphae were observed with a Nikon 80i fluorescence microscope (Nikon, Tokyo, Japan).

### 4.7. Virulence Assay

To evaluate FHB symptoms on wheat, two florets of a single central spikelet were point-inoculated at the mid-anthesis stage with a 10-μL solution comprising 1 × 10^3^ *F. graminearum* conidia in distilled water. The inoculated spikes were sprayed with water and wrapped in plastic film for 48 h to maintain humidity. The wheat plants were incubated at 25 °C in a controlled-environment room. Head blight symptoms were assessed at 2–12 days after inoculation, with 5–10 plants per treatment. To assess fungal pathogenicity in *A. thaliana*, the middle parts of leaves that had been wounded with a brush were point-inoculated with 1 × 10^3^ conidia. The inoculated leaves were maintained on Murashige and Skoog medium at 25 °C and then scanned with the EPSON Perfection V700 Photo Scanner.

To test whether *FgNahG* influences DON production in liquid medium, a two-stage protocol was completed [16,50]. Specifically, 1 × 10^4^ *F. graminearum* conidia were used to inoculate 5 mL first-stage medium (GYEP) in each well of six-well cell culture trays (Costar^®^, Corning, NY, USA). The trays were incubated at 28 °C on an orbital shaker (180 rpm) for 24 h in darkness. Following the 24-h growth period, the mycelial solids in each well were transferred to a 15-mL tube (Costar^®^) and washed with 10 mL distilled water. Next, 0.2 mg mycelia were transferred to a new well containing 4 mL second-stage medium (pH 4.0), after which 1 mM SA was added to evaluate the effect of SA on DON production. The medium was collected after 24 h and the DON concentration was measured with the DON ELISA kit (Beacon, Saco, ME, USA) and the Multiskan Spectrum microplate spectrophotometer (Thermo Scientific, Vantaa, Finland).

To assess whether *FgNahG* affects DON production on wheat plants, two florets from each fully developed spikelet of a whole spike at the mid-anthesis stage were inoculated with 1 × 10^3^ conidia. The inoculated spikes were treated as described above and were harvested at 6 days post-inoculation. The collected material was ground to a fine powder in liquid nitrogen, after which DON was extracted from 100 mg (fresh weight) spikelet powder with 1 mL distilled water and an incubation at 4 °C for 12 h. The analysis involved three biological replicates, with at least 10 heads per treatment. The DON content was measured as described above.

### 4.8. Quantitative Real-Time PCR Analyses

To prepare mycelial samples, mSNA medium in plates was inoculated with 1 × 10^3^ conidia and then maintained at 25 °C for 5 days before the resulting mycelia were harvested. To prepare spike samples, two florets from each fully developed spikelet in a whole spike at the mid-anthesis stage were inoculated with 1 × 10^3^ conidia. The inoculated wheat plants were incubated as described above and spike samples were harvested at 6, 12 and 24 hpi. Additionally, *A. thaliana* leaves were harvested at 30 days after germination for a subsequent RNA extraction. All harvested samples were ground to a fine powder in liquid nitrogen. Total RNA was extracted from 100 mg fresh tissue using the E.Z.N.A.^®^ Total RNA Kit I (Omega Bio-Tek, Norcross, GA, USA). The extracted RNA served as the template for a reverse transcription with the PrimeScript™ RT Reagent Kit with genomic DNA Eraser (Takara, Dalian, China).

The Rj-*FgNahG*-F and Rj-*FgNahG*-R primer pair was used to analyze the *FgNahG* expression levels in *F. graminearum* and *A. thaliana*. Genes encoding glyceraldehyde 3-phosphate dehydrogenase (*FgGAPDH*; *FG05_06257*), β-tubulin (*FG05_09530*) and elongation factor 1 (*FG05_08811*) were used as references for normalizing the quantitative real-time PCR (qRT-PCR) data (Table 1; [16]). Moreover, the relative abundance of *F. graminearum* in wheat spikes was estimated by measuring the *FgGAPDH* expression levels, with the qRT-PCR data normalized against the expression data for three wheat reference genes [*w-GAPDH* (wheat glyceraldehyde-3-phosphate dehydrogenase gene), NCBI UniGene Ta.66461; *Aox* (aldehyde oxidase gene), Ta.6172; and *hn-RNP-Q* (heterogeneous nuclear ribonucleoprotein Q gene), Ta.10105] [16]. The qRT-PCR analyses were completed with the MyiQ Real-Time PCR Detection System (Bio-Rad, Hercules, CA, USA).

### 4.9. Quantification of SA in Wheat Spikes and in A. thaliana Leaves

To prepare wheat spike samples, two florets from each fully developed spikelet in a whole spike at the mid-anthesis stage were inoculated with 1 × 10^3^ conidia. The inoculated wheat plants were treated as described above. At 6, 12 and 24 hpi, the spikes were harvested and ground to a fine powder in liquid nitrogen. Moreover, the *A. thaliana* samples used for the qRT-PCR assay were also applied for the SA measurement. Analyses were completed with three biological replicates per treatment. The SA was quantified as described by Siciliano et al. [51].

### 4.10. Statistical Analysis

Student’s *t*-test implemented with the DPS (Data Procession System) program (version 12.01, Zhejiang University, Hangzhou, China) was used to test the significance of the differences between treatments.

## Figures and Tables

**Table 1:** Primers used in this study. Restriction enzyme cut sites are underlined.

Primer	Sequence (5′–3′)	Reference
*FgNahG*-up-F	GGAAGCTTCACCCACTTTCTCACCA	this study
*FgNahG*-up-R	GGACTAGTCGAACTTTCTTCCGTATTG	
*FgNahG*-down-F	GCGGGCCCATCAGGATAGTAAACAGAA	this study
*FgNahG*-down-R	GCGAGCTCCGACTGGTTCCACTAC	
*∆*J-up-F	TTTGAGCGGCATTCTTG	this study
*∆*J-up-R	TGTTTCGGCGTGGGTA	
*∆*J-down-F	CAGTTGCCTAAATGAACC	this study
*∆*J-down-R	CTATCGTCAACGGGTCT	
R-*FgNahG* -F	CGAGCTCGAGGATCTGCCACAACAC	this study
R-*FgNahG* -R	AACTGCAGGATTGCTTCCAACAAACT	
30a-*FgNahG*-F	GGGGTACCATCATTGGAGCTGGCCTCTCA	this study
30a-*FgNahG*-R	ACGAGCTCTCAATTGCTTCCAACAAAC	
Fg-*GAPDH*-F	TGACTTGACTGTTCGCCTCGAGAA	[16]
Fg-*GAPDH*-R	ATGGAGGAGTTGGTGTTGCCGTTA	
Fg-*β-tubulin*-F	GTTGATCTCCAAGATCCGTG	[16]
Fg-*β-tubulin*-R	CATGCAAATGTCGTAGAGGG	
Fg-*Factor1*-F	CCTCCAGGATGTCTACAAGA	[16]
Fg-*Factor1*-R	CTCAACGGACTTGACTTCAG	
*Rj-FgNahG-F*	AGAAGGGAAGGCAGGAGCG	this study
*Rj-FgNahG-R*	TCATTCAAATCAACCGAGTAAAGC	
*ACT2-F*	CTTGCACCAAGCAGCATGAA	[42]
*ACT2-R*	CCGATCCAGACACTGTACTTCCTT	
*Aox*-F	GACTTGTCATGGTAGATGCCTG	[16]
*Aox*-R	CAGGACGAGCATAACCATTCTC	
w-*GAPDH*-F	AACTGTTCATGCCATCACTGCCAC	[16]
w-*GAPDH*-R	AGGACATACCAGTGAGCTTGCCAT	
*hn-RNP-Q*-F	TCACCTTCGCCAAGCTCAGAACTA	[16]
*hn-RNP-Q*-R	AGTTGAACTTGCCCGAAACATGCC	

## Supplementary Materials

The following are available online at http://www.mdpi.com/2072-6651/11/2/59/s1, Figure S1: Levels of JA (jasmonic acid) in spikes inoculated with water (CK treatment), the WT (wild type) strain or the Δ*FgNahG* strain at 6, 12 and 24 h after inoculations. Values are provided as the mean ± standard deviation of three biological replicates per treatment. Different letters above each column indicate significant differences at *p* ≤ 0.05. FW, fresh weight.
