# Supplementary material for: Functional Analysis of FgNahG Clarifies the Contribution of Salicylic Acid to Wheat (Triticum aestivum) Resistance against Fusarium Head Blight
Source: Toxins (Basel). 2019 Jan 22;11(2):59. doi: 10.3390/toxins11020059 (PMC6410203; doi:10.3390/toxins11020059)
Supplement: Supplementary file 1 [file toxins-11-00059-s001.pdf]

Supplementary Materials: Functional Analysis of *FgNahG* Clarifies the Contribution of Salicylic Acid to Wheat (*Triticum aestivum*) Resistance against Fusarium Head Blight

Peng-Fei Qi, Ya-Zhou Zhang, Cai-Hong Liu, Qing Chen, Zhen-Ru Guo, Yan Wang, Bin-Jie Xu, Yun-Feng Jiang, Ting Zheng, Xi Gong, Cui-Hua Luo, Wang Wu, Li Kong, Mei Deng, Jian Ma, Xiu-Jin Lan, Qian-Tao Jiang, Yu-Ming Wei, Ji-Rui Wang and You-Liang Zheng

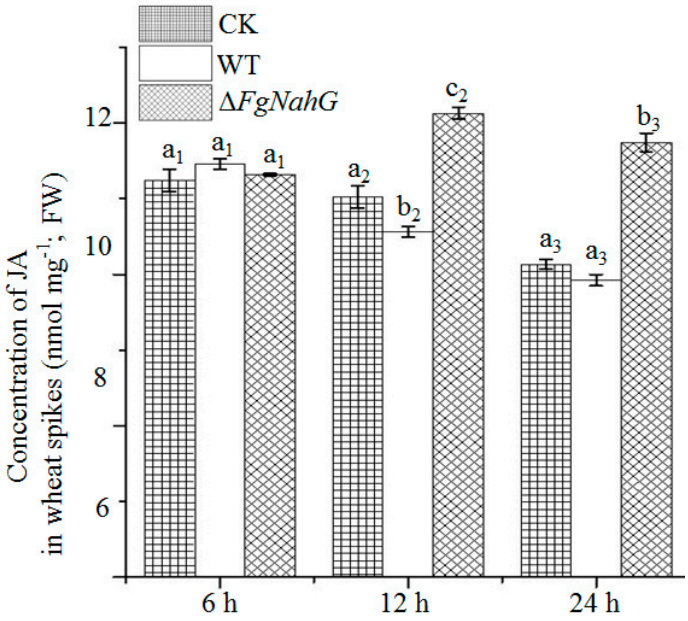

Figure S1. Levels of JA (jasmonic acid) in spikes inoculated with water (CK treatment), the WT (wild type) strain or the Δ*FgNahG* strain at 6, 12 and 24 h after inoculations. Values are provided as the mean ± standard deviation of three biological replicates per treatment. Different letters above each column indicate significant differences at $p \leq 0.05$. FW, fresh weight.
